# Research Trends in Sport Pedagogy and Physical Education: A Bibliometric Analysis of Motivation, Self-Efficacy, Creativity, and Critical Thinking

**DOI:** 10.3390/bs16091673

**Published:** 2026-09-17

**Authors:** Laura Ruiz Sánchez, Andrea Ceciliani, Gabriele Russo, Ramón Chacón-Cuberos

**Affiliations:** 1 Department of Didactic of Musical, Plastic, and Corporal Expression, University of Jaén, 23071 Jaén, Spain; 2 Dipartimento di Scienze per la Qualità della Vita, University of Bologna, 47921 Rimini, Italy; 3 Department of Economics, Engineering, Society and Business Organization (DEIM), University of Tuscia, 01100 Viterbo, Italy; 4 Department of Research Methods and Diagnosis in Education, University of Granada, 18071 Granada, Spain

**Keywords:** sport pedagogy, physical education, motivation, self-efficacy, creativity, critical thinking, positive psychology, bibliometric analysis

## Abstract

Contemporary sport pedagogy increasingly emphasises learner-centred, inclusive, and psychologically supportive approaches. This bibliometric review mapped research at the intersection of Physical Education, well-being-related perspectives, and motivation, self-efficacy, creativity, and critical thinking. Scopus and the Web of Science Core Collection were searched, and the records were processed in R (version 4.5.0) using the bibliometrix package (version 5.3.0) and its Biblioshiny web interface. The searches identified 683 records; after 208 initial duplicates were removed, all 475 unique records were re-screened. A further eight residual duplicates and 261 ineligible records were removed, yielding 206 documents published between 2008 and 2026 across 95 sources. The corpus averaged 16.35 citations per document and 26.7% international co-authorship. Motivation was explicitly represented in 174 documents (84.5%), self-efficacy in 34 (16.5%), creativity in 16 (7.8%), and critical thinking in four (1.9%). A 58-node author-keyword network and a five-cluster thematic map showed substantially greater consolidation around motivation and self-determination theory than around creativity or critical thinking. These findings describe the retrieved intersectional corpus rather than the whole Physical Education literature.

## 1. Introduction

### 1.1. Evolution of Physical Education

Physical Education has progressively expanded its educational scope beyond the acquisition of motor skills and the improvement of physical fitness. Within contemporary sport pedagogy, school-based Physical Education is increasingly understood as a context for promoting students’ cognitive, emotional, social and physical development through meaningful bodily experiences (Casey & Goodyear, 2015; Metzler, 2017; Russo et al., 2025). This shift reflects a broader movement towards student-centred, inclusive and competency-based approaches in which learning also involves decision-making, autonomy, cooperation, reflection and continued engagement in physical activity (Casey et al., 2017; Dyson, 2002).

In this context, motivation has become one of the most extensively examined constructs in Physical Education research. Self-determination theory proposes that the satisfaction of autonomy, competence, and relatedness supports self-determined forms of motivation (Deci & Ryan, 1985; Ryan & Deci, 2000). In Physical Education, students who perceive greater autonomy, competence, and social connection are more likely to report intrinsic motivation, enjoyment, effort, persistence, and positive attitudes towards physical activity (Ntoumanis, 2001; Standage et al., 2005; Wallhead & Ntoumanis, 2004). A systematic review and meta-analysis of 265 studies found that satisfaction of these psychological needs was strongly associated with autonomous motivation, which in turn was related to adaptive educational outcomes (Vasconcellos et al., 2020). Motivation is therefore central to both immediate engagement in lessons and students’ longer-term relationship with active lifestyles.

### 1.2. Creativity, Critical Thinking, Self-Efficacy, and Motivation in Physical Education

Self-efficacy is another key construct for explaining students’ participation and learning in Physical Education. According to Social Cognitive Theory, self-efficacy refers to individuals’ beliefs in their ability to organise and execute the actions required to achieve specific goals (Bandura, 1977). In educational and physical activity contexts, stronger self-efficacy beliefs are associated with greater confidence, effort, persistence, and recovery from failure (Bandura, 1977; Moritz et al., 2000). Within Physical Education, perceived competence and motor self-efficacy are closely related to students’ willingness to participate and confidence in learning new skills (Gao et al., 2008). Consequently, self-efficacy can be understood as a psychological mechanism linking pedagogical experiences with behavioural, emotional, and motor outcomes.

Beyond motivation and self-efficacy, creativity and critical thinking are increasingly relevant for rethinking Physical Education as a space for complex and meaningful learning. Creativity can be understood as the capacity to generate ideas or actions that are both original and appropriate within a given context (Beghetto & Kaufman, 2007; Runco & Jaeger, 2012). In Physical Education, it may be expressed through movement exploration, tactical solutions, bodily expression, problem solving, and the generation of alternative motor responses. Critical thinking involves purposeful, reflective, and reasoned judgement that supports decision-making and problem solving (Halpern, 1998). Applied to Physical Education, it enables students to analyse tactical situations, reflect on performance, make informed decisions about health and physical activity, and interpret movement from a broader educational perspective. Although both constructs are central to contemporary education, their integration into Physical Education research remains less consolidated than motivational approaches.

### 1.3. Positive Psychology and Physical Education

Positive Psychology provides a potentially useful interpretive lens for considering these variables within a holistic understanding of Physical Education, but it should not be assumed to be the field’s empirically dominant framework. Rather than focusing exclusively on deficits or performance outcomes, positive psychological perspectives examine strengths, positive emotions, engagement, relationships, meaning, accomplishment, and well-being (Seligman & Csikszentmihalyi, 2000; Seligman et al., 2009). In educational settings, these perspectives have informed approaches to well-being, resilience, engagement, social connection, and personal growth (Kern et al., 2015; Norrish et al., 2013; Waters, 2011). Physical Education offers a relevant context for this lens because it combines embodied learning, interpersonal interaction, emotional experience, challenge, achievement, and self-perception.

Existing reviews have addressed game-centred pedagogies and emotional intelligence in Physical Education (Harvey & Jarrett, 2014; Rico-González, 2023), while other bodies of work have examined motivation, perceived competence, creativity, and social-emotional learning separately. This separation suggests a need to examine whether and how these lines are connected in the bibliographic record. To our knowledge, previous reviews have not jointly mapped motivation, self-efficacy, creativity, and critical thinking within a corpus explicitly intersecting Physical Education with positive psychological and well-being terminology.

Bibliometric analysis is suitable for this purpose because it supports systematic examination of scientific production, citation visibility, collaboration, and conceptual structures (Aria & Cuccurullo, 2017; Donthu et al., 2021; Zupic & Čater, 2015). Citation counts are treated here as indicators of bibliometric visibility rather than direct evidence of pedagogical influence or study quality.

Accordingly, this study aimed to describe international research retrieved at the intersection of Physical Education, well-being-related or positive psychological terminology, and motivation, self-efficacy, creativity, or critical thinking. Four research questions guided the analysis: RQ1, how has scientific production evolved over time? RQ2, which countries, institutions, sources, authors, and globally cited documents are most visible in the retrieved corpus? RQ3, how are the four focal constructs represented and how has that representation evolved? RQ4, what conceptual and thematic structures emerge from author-keyword co-occurrence?

## 2. Materials and Methods

### 2.1. Study Design

This study was designed as a descriptive and retrospective bibliometric analysis of research retrieved at the intersection of Physical Education, positive psychological or well-being-related terms, and the four focal constructs. Performance analysis and science mapping were combined to describe productivity, bibliometric visibility, collaboration, and conceptual structure (Aria & Cuccurullo, 2017; Donthu et al., 2021; Zupic & Čater, 2015).

### 2.2. Data Sources

The bibliographic records were retrieved from Scopus and the Web of Science Core Collection, two of the most widely used multidisciplinary databases for bibliometric research due to their broad coverage of peer-reviewed scientific literature and structured citation metadata. The use of both databases allowed for a more comprehensive retrieval of publications and reduced the risk of database-specific bias. Records were exported in BibTeX format, including titles, abstracts, authors, affiliations, keywords, source titles, publication years, citation counts, document types, and digital object identifiers when available.

### 2.3. Search Strategy

The search strategy deliberately targeted an intersection rather than the entire Physical Education literature. Terms were organised into three mandatory conceptual blocks: (a) Physical Education; (b) Positive Psychology and well-being-related terms; and (c) creativity, critical thinking, self-efficacy, or motivation. The AND operator connected the three blocks; therefore, findings are interpreted only for the corpus produced by this intersectional query.

The conceptual structure of the search strategy is presented in Table 1.

After defining the conceptual blocks of the search strategy, the final search equation was adapted to the specific syntax and search fields of each database. In Scopus, the search was conducted using the TITLE-ABS-KEY field, whereas in Web of Science Core Collection, the Topic Search field was used. The database-specific adaptations of the search strategy are presented in Table 2.

The searches were finalised on 14 May 2026. They were limited to documents published in English or Spanish, with no geographical restrictions. All publication years available up to the search date were considered; the earliest document retained in the final corpus was published in 2008. The 20 records indexed for 2026 were retained in the corpus-level analyses, but 2026 was omitted from temporal-trend figures because the publication year was incomplete.

### 2.4. Inclusion and Exclusion Criteria

Documents were included when they: (a) were peer-reviewed journal articles or reviews indexed in Scopus or the Web of Science Core Collection; (b) examined school Physical Education, university Physical Education classes, or initial Physical Education teacher education; (c) contained a direct Physical Education teaching, learning, subject-specific, or teacher-education component; (d) contained at least one term from the Positive Psychology/well-being block and at least one term from the focal-construct block, consistent with the three mandatory search blocks; and (e) were published in English or Spanish. Mixed-subject studies were retained only when they reported separable, subject-specific Physical Education measures or results.

Documents were excluded when they focused on general physical activity without an instructional Physical Education context; elite or recreational sport, athletes, coaching, or performance without educational Physical Education; clinical exercise, rehabilitation, patient education, or therapy; general education or general teacher education without a direct Physical Education component; or ineligible publication types, retracted items, or duplicates. University studies were retained only when they concerned university Physical Education courses or initial Physical Education teacher education.

### 2.5. Document Selection Process

**Figure 1:**
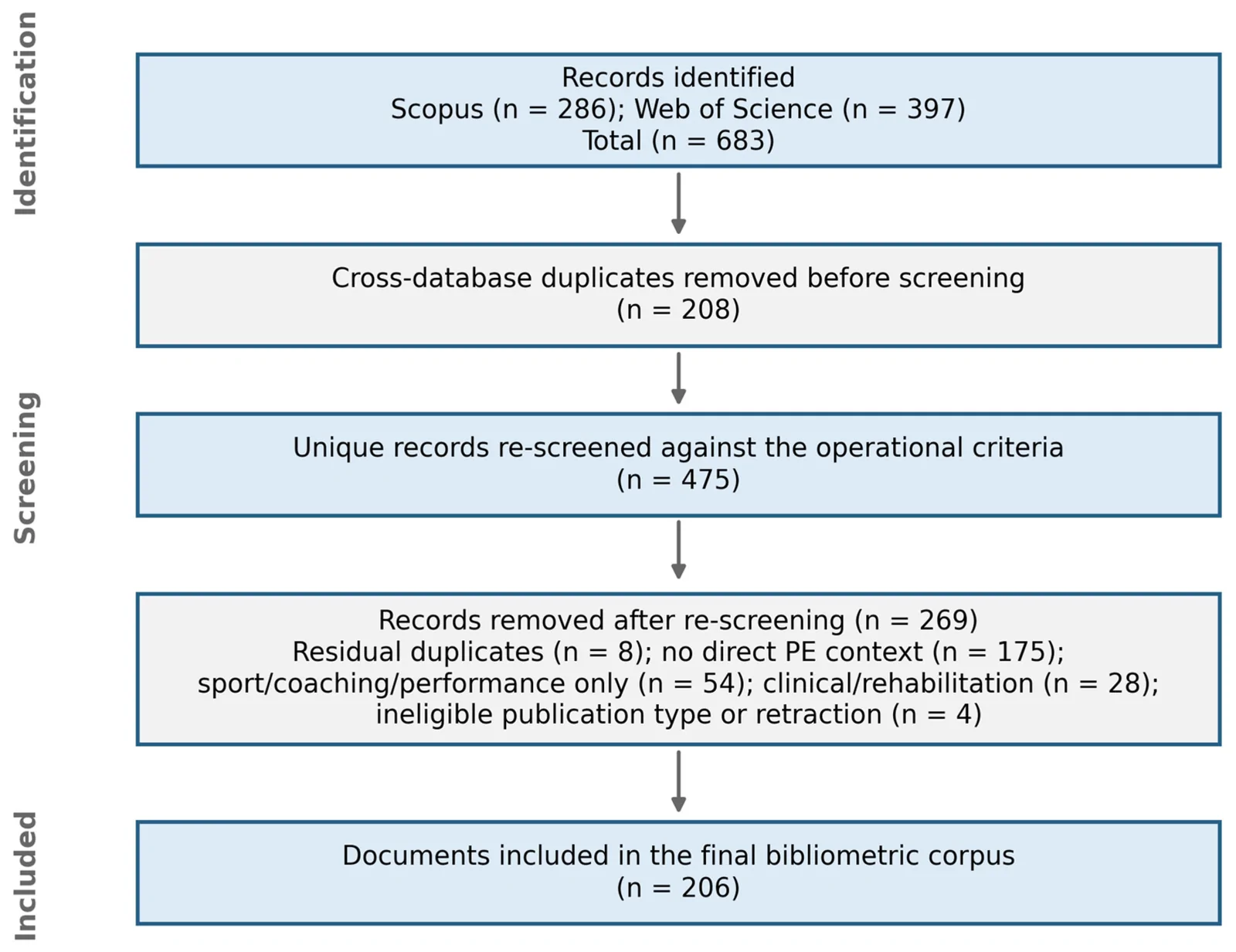
PRISMA-style flow of identification, re-screening, exclusion, and final inclusion. Blue boxes indicate records retained in the selection process, whereas grey boxes indicate records removed or excluded.

The searches identified 286 Scopus and 397 Web of Science records (*n* = 683). Removal of 208 cross-database duplicates yielded 475 records. In response to concerns about corpus validity, all 475 records were re-screened using a documented decision codebook and the revised operational criteria. The eligibility audit was conducted independently by two members of the research team (L.R.S. and R.C.-C.), who assessed the complete set of records against the predefined eligibility criteria and decision codebook. Following these independent assessments, records considered uncertain or proposed for exclusion were reviewed collectively by the research team. Any discrepancies or borderline cases were discussed until consensus was reached on the final eligibility decision. Titles, abstracts, keywords, sources, document types, and DOIs were checked; full texts were consulted whenever metadata were insufficient. Borderline records were subsequently subjected to a targeted audit of whether Physical Education was the instructional context rather than an incidental mention. The complete procedure identified 8 residual duplicates and excluded 261 records: 175 without a direct Physical Education context, 54 limited to sport/coaching/performance, 28 clinical or rehabilitation records, and 4 ineligible publication types or retracted items. The final corpus comprised 206 documents (Figure 1). A complete list and machine-readable datasets are provided in Files S1–S4.

### 2.6. Data Extraction and Cleaning

The final dataset contained titles, abstracts, authors, affiliations, countries, author keywords, Keywords Plus, source titles, years, citation counts, document types, cited references, and DOIs when available. Screening decisions were linked to record identifiers and reasons for exclusion. Keyword cleaning used a manually documented thesaurus: wellbeing, health and well-being, and overall well-being were merged into well-being; singular/plural variants were harmonised for adolescent(s), teacher(s), student(s), and child(ren); sports was merged into sport; PE into Physical Education; self-determination into self-determination theory; emotion into emotions; and primary schools into primary school. Malformed or non-informative terms (physical, basic, and theory) were removed. The raw and cleaned keyword fields were retained for traceability.

### 2.7. Bibliometric Analysis

Performance indicators comprised annual production, sources, authors, collaboration, corresponding-author countries, institutional affiliation occurrences, and global citations within the retrieved corpus. Country collaboration was described using single-country publications (SCP) and multiple-country publications (MCP). The annual growth-rate statistic was not reported because the endpoint year was incomplete and the early time series was sparse. Global and normalised citation counts were treated as bibliometric visibility indicators and were not interpreted as direct measures of study quality or Physical Education-specific pedagogical influence.

Science mapping used author keywords (DE) only. Terms occurring in at least three documents (1.46% of the corpus) were retained, producing a 58-node co-occurrence network. Edge weights were normalised by association strength; the Fruchterman–Reingold layout and Louvain community detection were applied with seed 1234. All 58 retained nodes were plotted, and node size reflected PageRank calculated on the raw co-occurrence network. The thematic map also used author keywords, a minimum frequency of three documents (Bibliometrix minfreq = 14 per thousand), unigrams, no stemming, two label terms per cluster, and alpha = 0.5. Themes were interpreted from their positions relative to median centrality and density.

Construct categories were non-exclusive and were operationalized using case-insensitive lexical families in titles, abstracts, author keywords, and Keywords Plus: motivat*; self-effic*; creativ*; and the phrase critical thinking. Explicit representation required a match in any of these four fields; the stricter salience measure required a match in the title or author keywords. Licencing strings containing ‘Creative Commons’ or ‘creativecommons’ were removed before creativity classification. The complete document-level classification is supplied as Supplementary Material File S4.

### 2.8. Software

Analyses were conducted in R 4.5.0 with Bibliometrix 5.3.0. Bibliometrix was used to process the records, calculate performance indicators, and generate the thematic map.

Python 3.12 was used to independently verify construct frequencies and the DE-only author-keyword network and to generate publication-quality figures; Microsoft Excel was used for screening verification and exported-output checks.

## 3. Results

### 3.1. General Overview of the Dataset

The searches retrieved 683 records. After 208 initial duplicates, eight residual duplicates, and 261 ineligible records were removed, 206 documents were included in the final analysis.

The corpus covered 2008–2026 and comprised 95 sources, 751 authors, and 883 author appearances. It included 183 articles, nine early-access articles, 13 reviews, and one early-access review. Eleven documents were single-authored; the mean was 4.29 co-authors per document and international co-authorship was 26.70%. The mean citation count was 16.35 per document, and Bibliometrix identified 8567 cited references.

### 3.2. Temporal Evolution of Scientific Production

**Figure 2:**
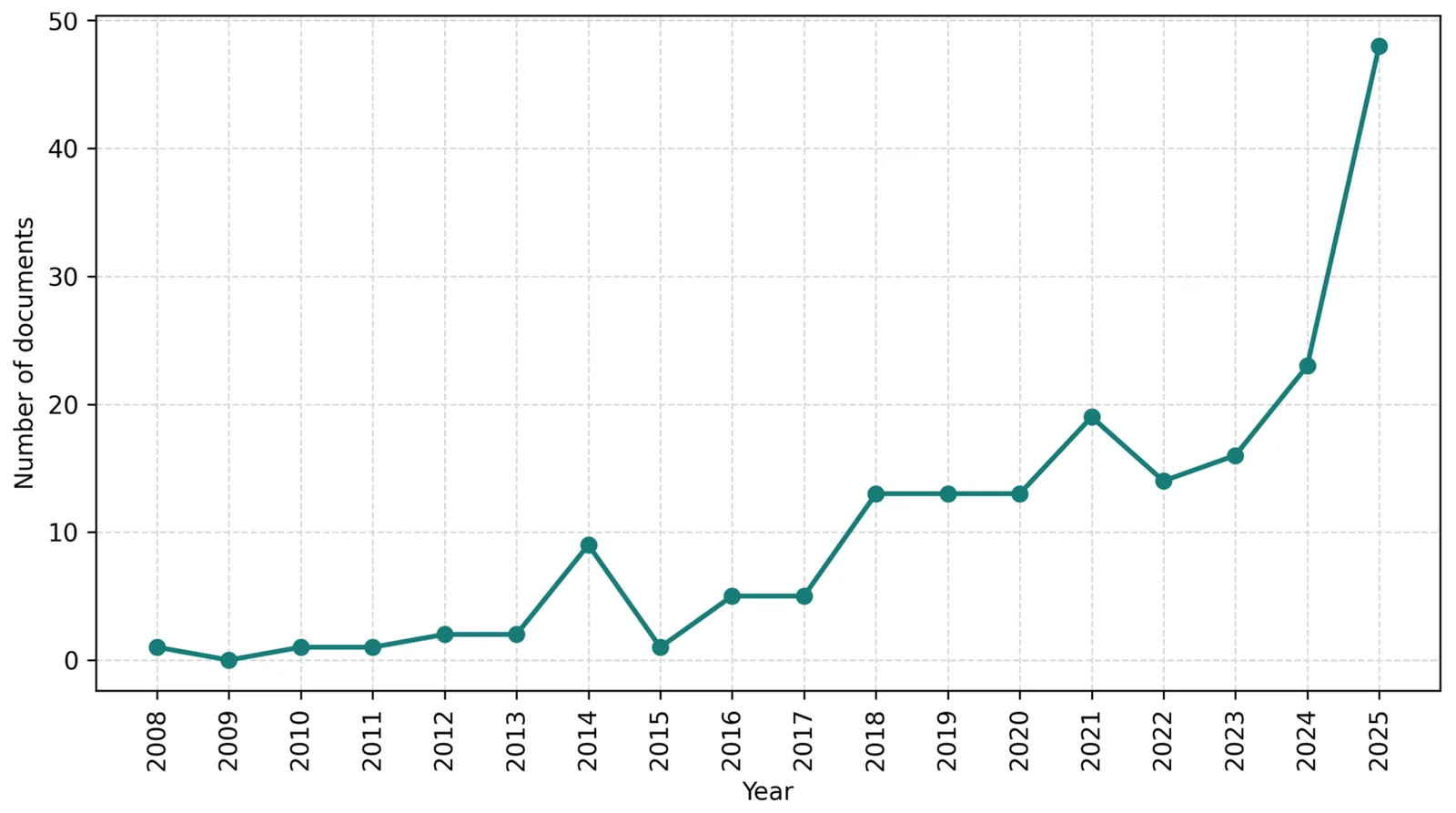
Annual scientific production through the last complete year (2025); zero-output years are shown.

Annual output was intermittent through 2013, increased to nine documents in 2014, and then rose more consistently (Figure 2).

Production reached 19 documents in 2021, 23 in 2024, and 48 in 2025. The 20 records from 2026 were retained for corpus-level analyses but omitted from Figure 2 because the search captured only part of that year.

### 3.3. Distribution by Countries and Institutions

**Figure 3:**
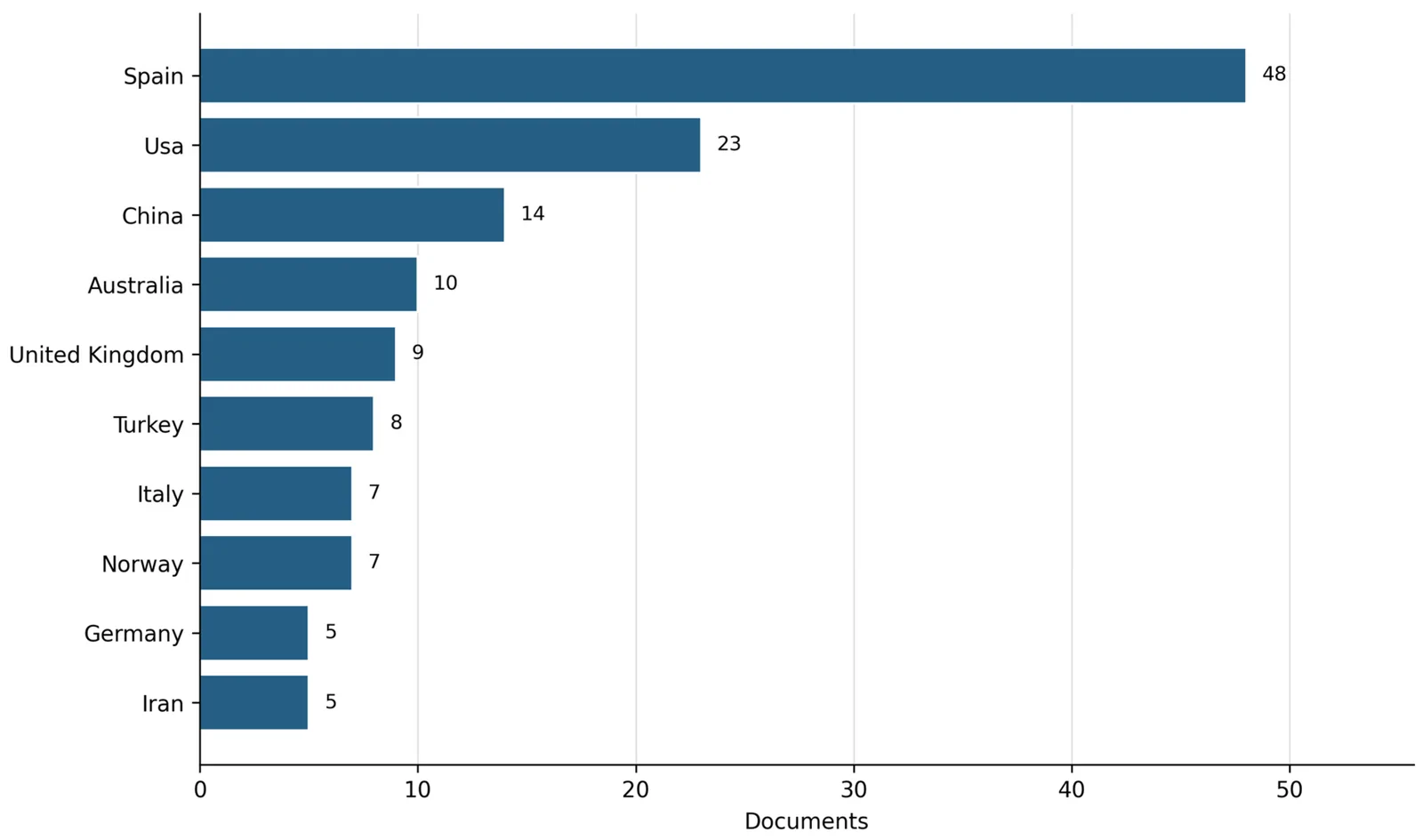
Leading countries by corresponding-author publication output.

Among the 202 documents with an identifiable corresponding-author country, Spain ranked first with 48 documents (23.8%), followed by the United States (23), China (14), Australia (10), and the United Kingdom (9) (Figure 3; Table 3).

Australia showed the highest MCP proportion among the ten leading countries (70.0%), followed by the United Kingdom (55.6%) and Turkey (50.0%). Spain combined the largest output with a lower MCP proportion (10.4%).

**Figure 4:**
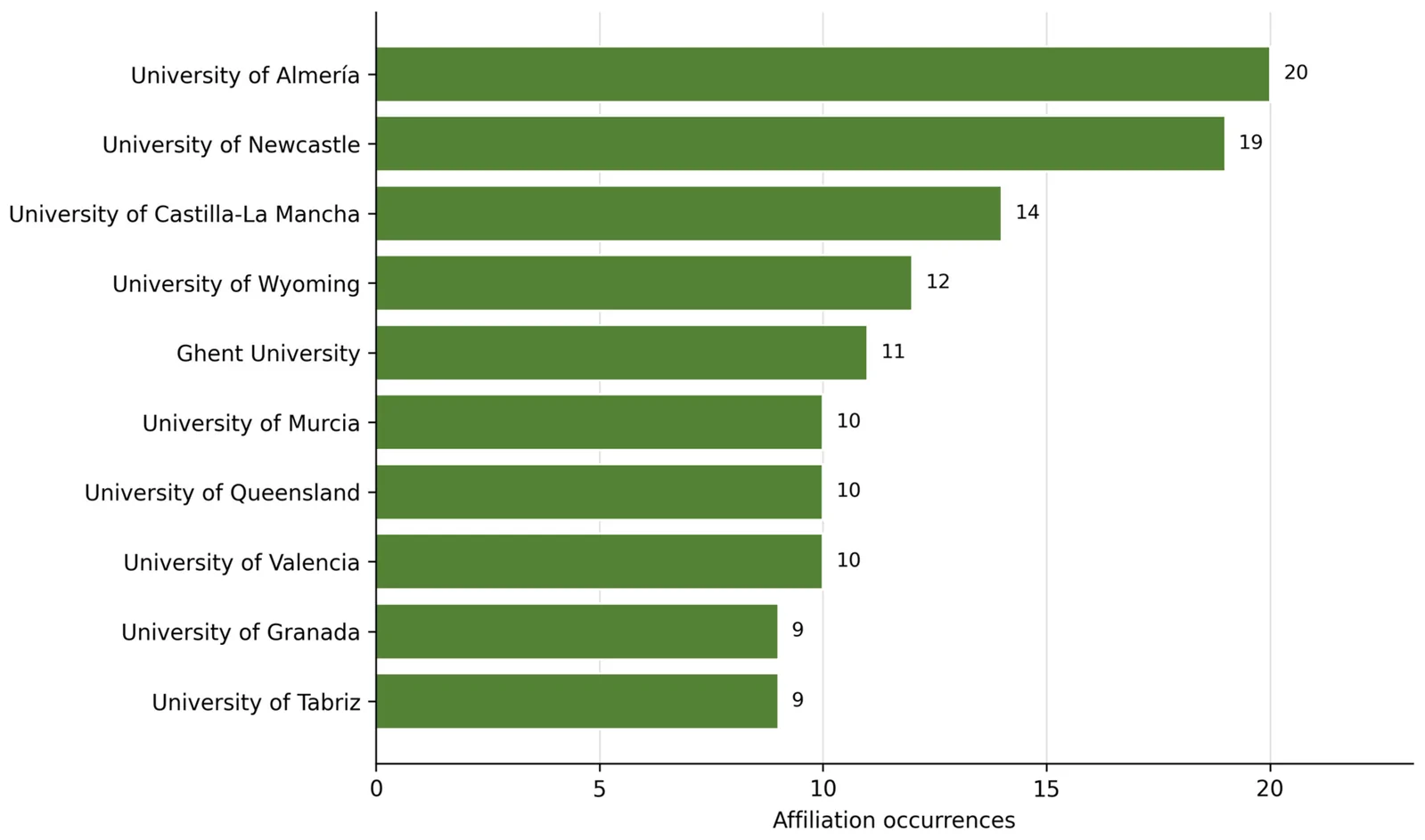
Most frequent institutional affiliations in the final corpus.

Institutional affiliation occurrences were led by the University of Almería (20), the University of Newcastle (19), and the University of Castilla-La Mancha (14) (Figure 4; Table 4). Because one paper may contain several authors from the same institution, these values describe affiliation occurrences rather than unique papers.

### 3.4. Most Relevant Journals

**Figure 5:**
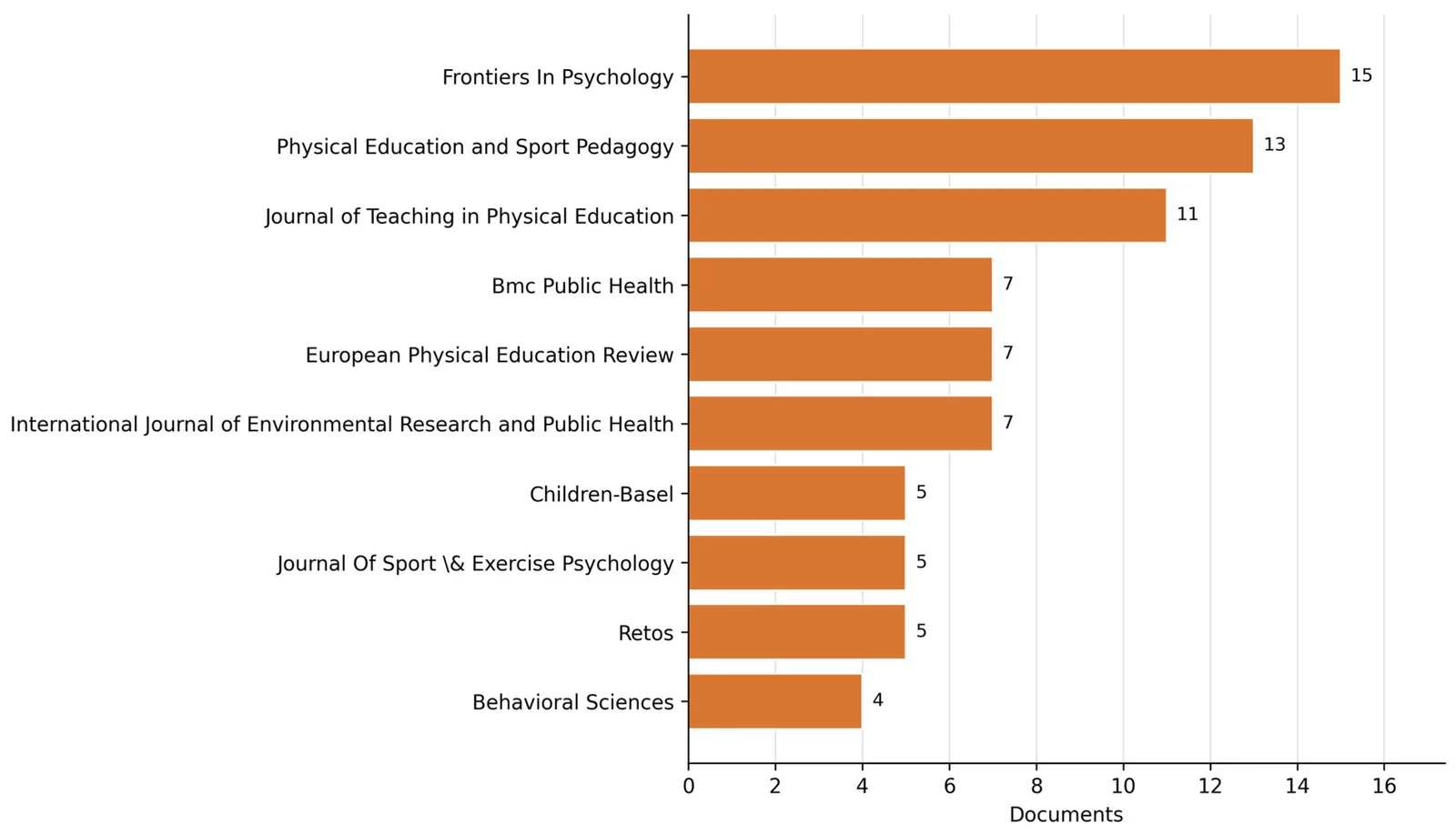
Most relevant sources by publication output.

The 206 documents appeared in 95 sources. Frontiers in Psychology led with 15 documents, followed by Physical Education and Sport Pedagogy (13), the Journal of Teaching in Physical Education (11), and three sources with seven documents each: BMC Public Health, the European Physical Education Review, and the International Journal of Environmental Research and Public Health (Figure 5; Table 5).

### 3.5. Author Productivity

**Figure 6:**
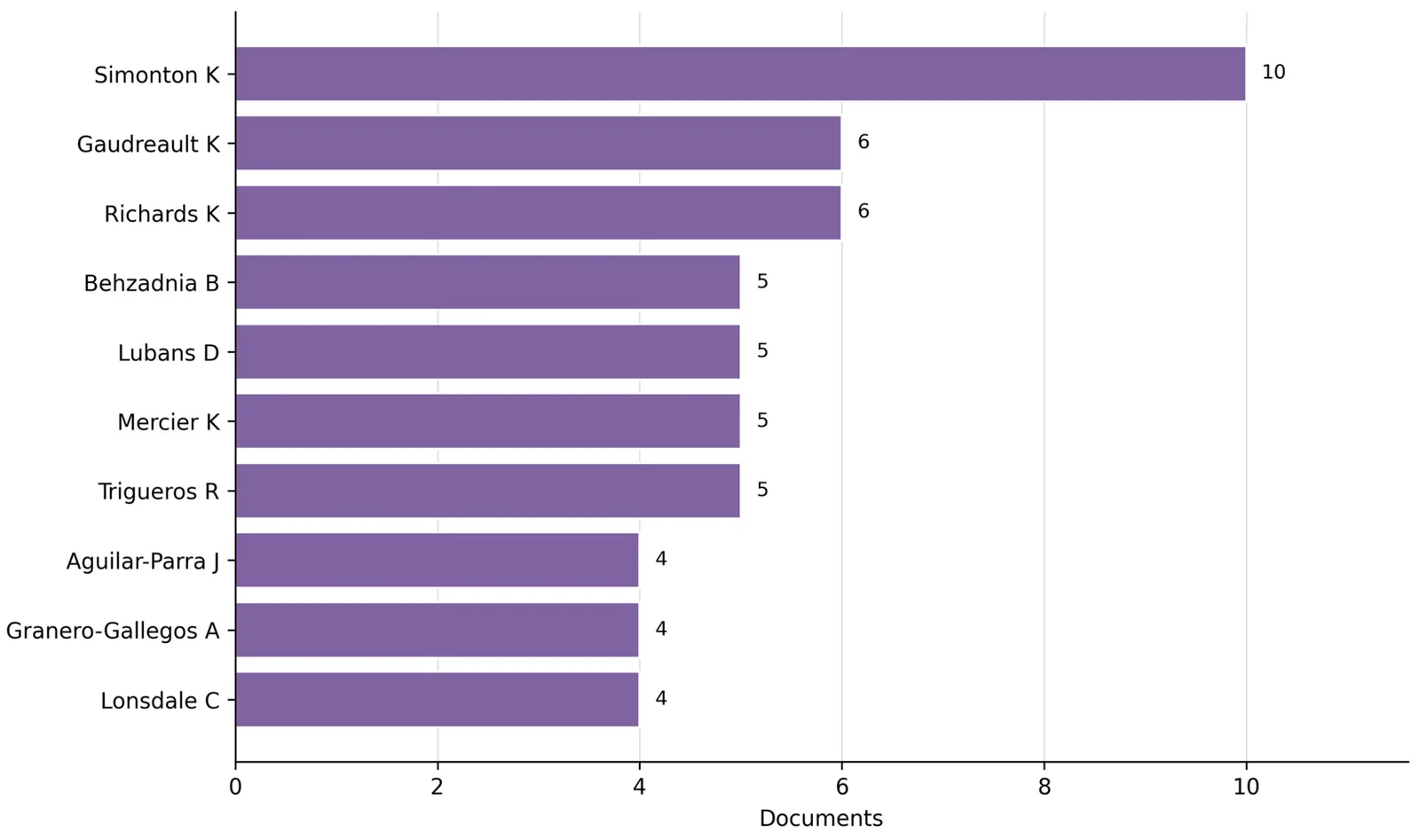
Most productive authors by number of documents.

Simonton K was the most productive author with 10 documents. Gaudreault K and Richards K each contributed six documents, while Behzadnia B, Lubans D, Mercier K, and Trigueros R each contributed five documents (Figure 6; Table 6).

Fractional counting was highest for Simonton K (2.47) and Behzadnia B (2.33), illustrating the effect of team size on author-level output.

### 3.6. Most Cited Documents

**Figure 7:**
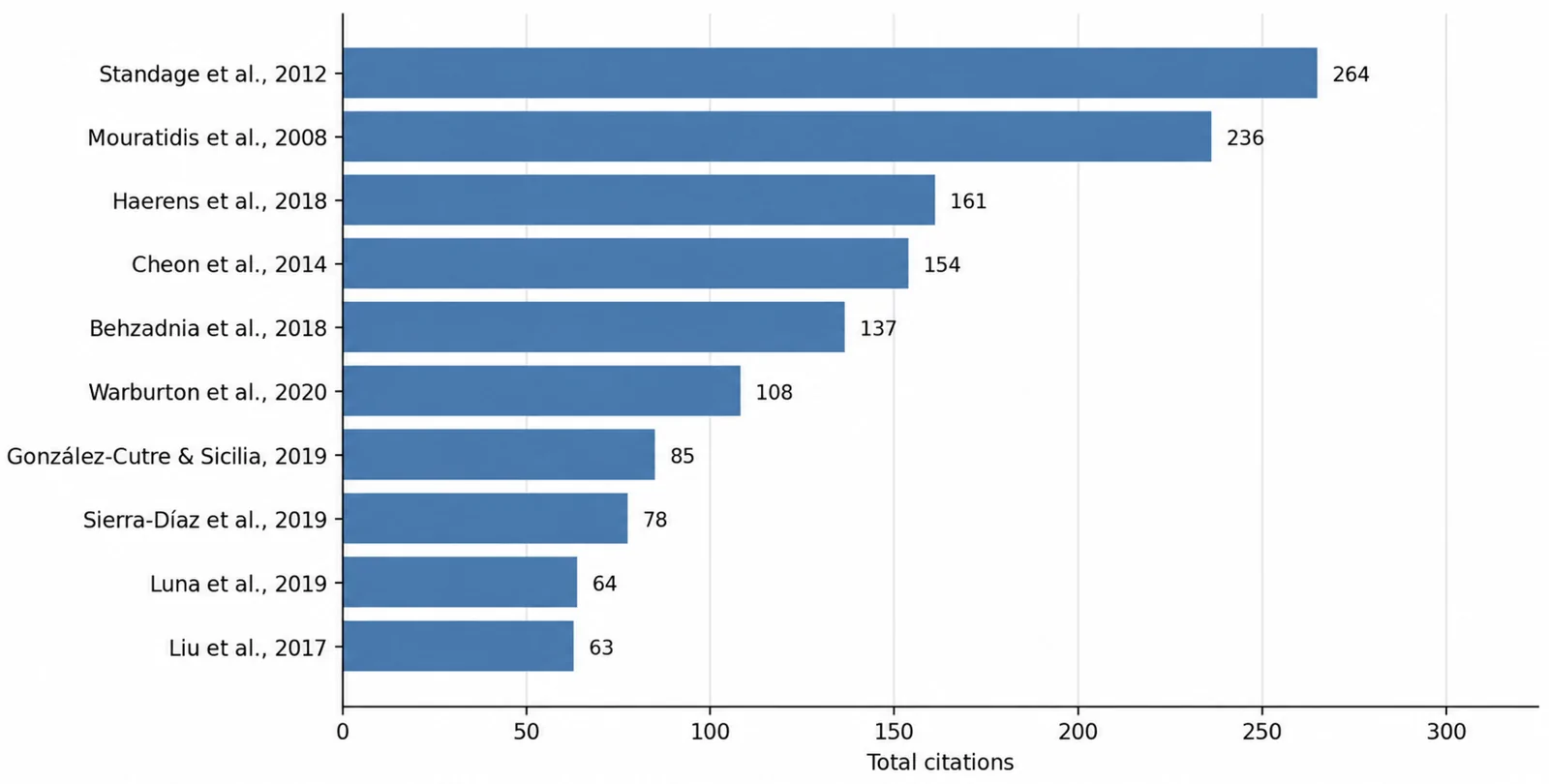
Global citation counts of the ten most-cited documents in the retrieved corpus (Standage et al., 2012; Mouratidis et al., 2008; Haerens et al., 2018; Cheon et al., 2014; Behzadnia et al., 2018; Warburton et al., 2020; González-Cutre & Sicilia, 2019; Sierra-Díaz et al., 2019; Luna et al., 2019; Liu et al., 2017).

The study by Standage et al. (2012) was the most globally cited document within the retrieved corpus, with 264 citations, followed by Mouratidis et al. (2008), with 236 citations, and Haerens et al. (2018), with 161 citations (Figure 7; Table 7).

The highest normalised citation score among the ten leading papers was recorded for Warburton et al. (2020), with a score of 4.63, followed by Haerens et al. (2018), with a score of 4.14.

### 3.7. Keyword Co-Occurrence Analysis

**Figure 8:**
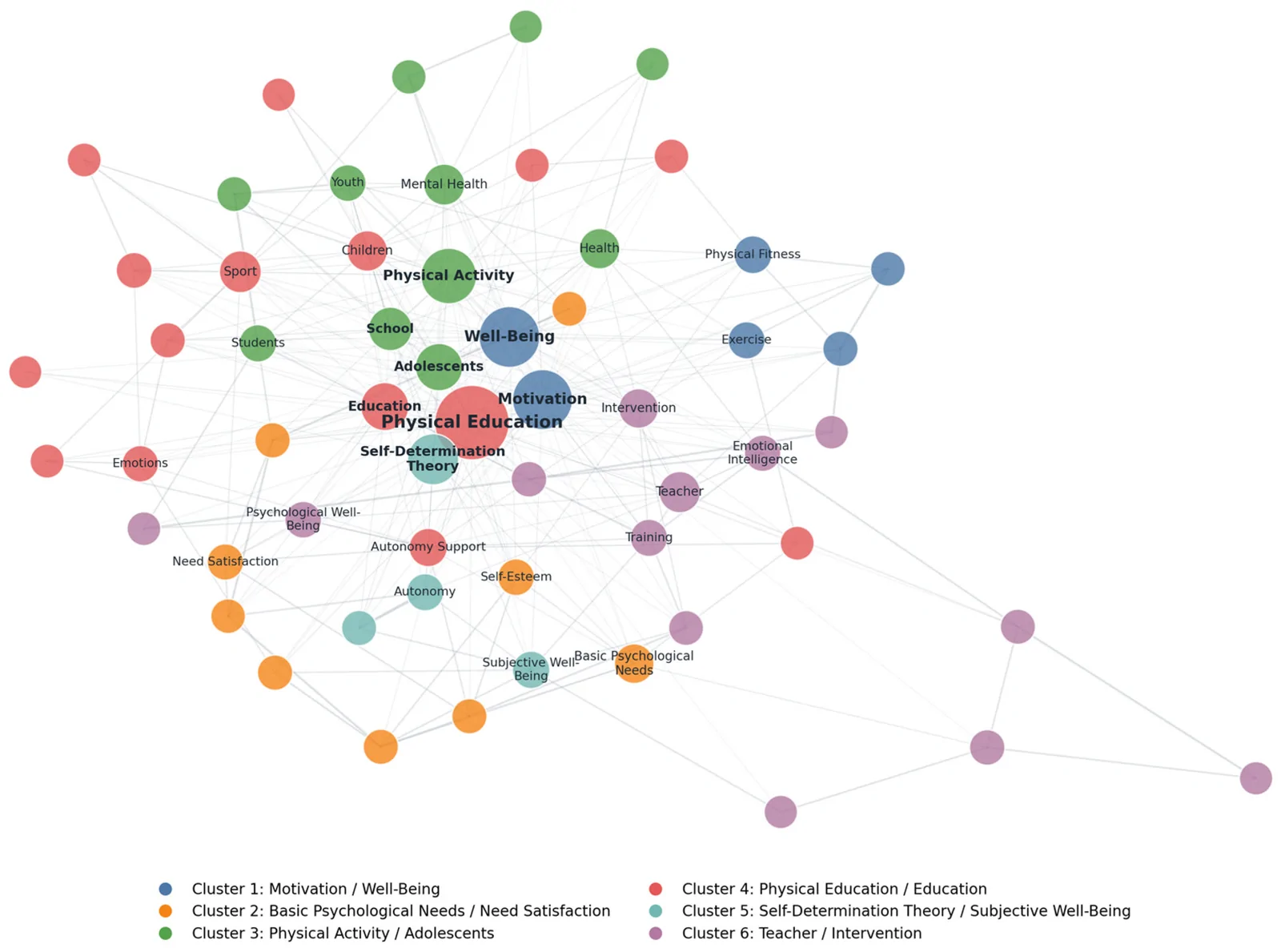
Author-keyword co-occurrence network based only on DE. Terms occurred in at least three documents; association-strength edge normalisation, Fruchterman–Reingold layout, Louvain clustering, and PageRank-scaled nodes were used. Labels are shown for the 28 leading nodes.

The 58-node DE-only author-keyword network identified Physical Education (PageRank = 0.122), Well-Being (PageRank = 0.074), Motivation (PageRank = 0.071), Physical Activity (PageRank = 0.057), and Self-Determination Theory (PageRank = 0.045) as the five leading terms (Figure 8).

Six communities were identified. Cluster 1 linked Motivation, Well-Being, Exercise, and Physical Fitness; Cluster 2 linked Basic Psychological Needs, Need Satisfaction, Self-Esteem, and Sport Education; Cluster 3 linked Physical Activity, Adolescents, School, and Mental Health; Cluster 4 linked Physical Education, Education, Children, and Sport; Cluster 5 linked Self-Determination Theory, Subjective Well-Being, Autonomy, and Competence; Cluster 6 linked Teacher, Intervention, Psychological Well-Being, and Teacher Well-Being.

Neither creativity nor critical thinking met the three-document author-keyword threshold, although both were detected by the broader construct classification.

### 3.8. Thematic Structure of the Research Field

**Figure 9:**
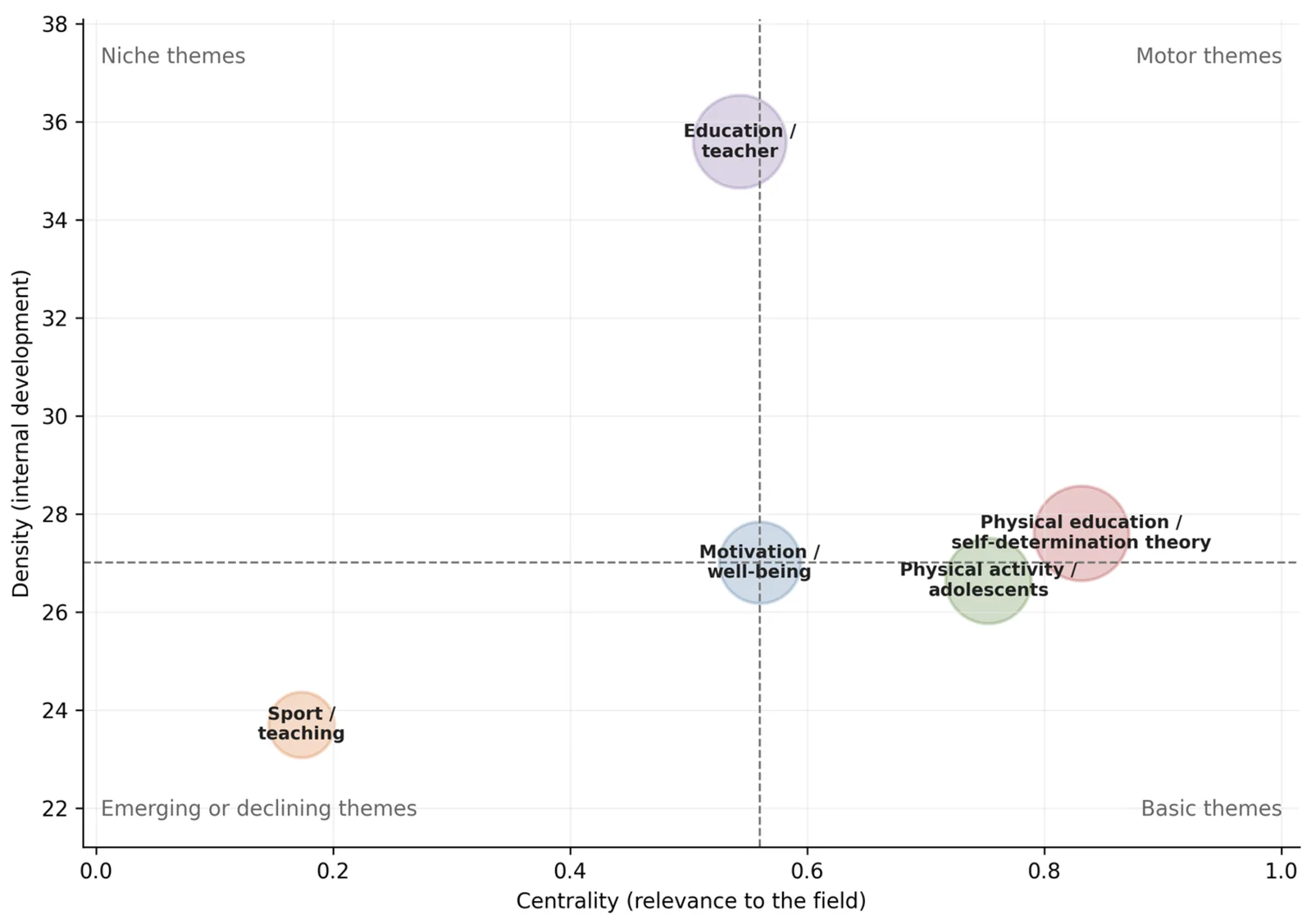
Thematic map based on author keywords; bubble size represents cluster frequency.

The thematic map produced five clusters (Figure 9). Physical Education/self-determination theory occupied the motor-theme quadrant; physical activity/adolescents formed a basic theme; and education/teacher formed a niche theme.

Motivation/well-being lay close to the median centrality-density boundaries, indicating a transversal but intermediate thematic position. Sport/teaching occupied the emerging or declining quadrant. Creativity and critical thinking did not form independent thematic clusters.

### 3.9. Construct-Specific Representation

**Figure 10:**
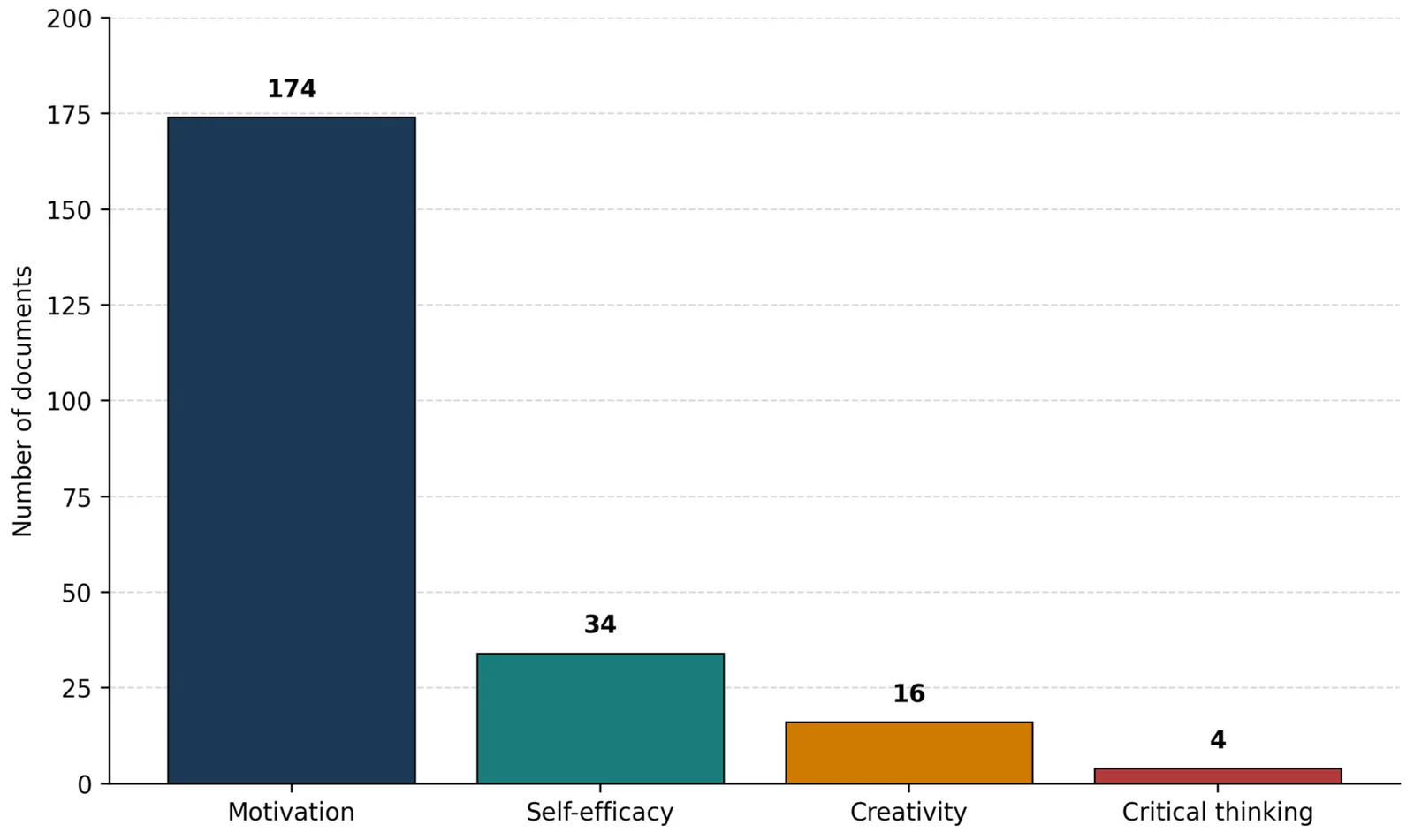
Explicit representation of the four focal constructs; categories overlap.

**Figure 11:**
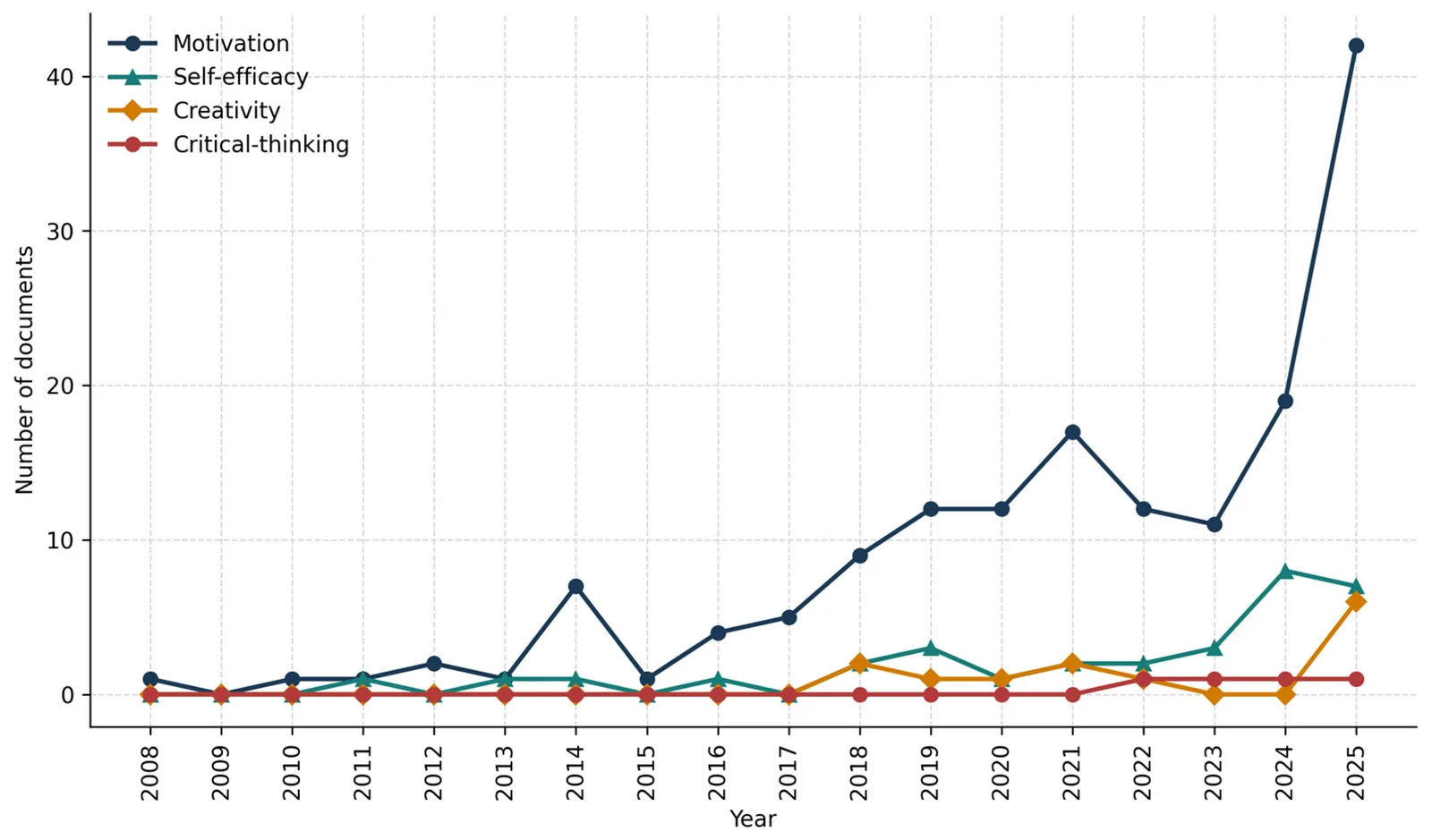
Annual evolution of the four focal constructs through 2025; zero-output years are shown.

The four construct categories were non-exclusive. Motivation was explicitly represented in 174 documents (84.5%), self-efficacy in 34 (16.5%), creativity in 16 (7.8%), and critical thinking in four (1.9%) (Table 8; Figure 10). A stricter salience measure based only on titles or author keywords yielded 76 documents for motivation (36.9%), 11 for self-efficacy (5.3%), seven for creativity (3.4%), and one for critical thinking (0.5%). Three included documents contained none of the four literal construct labels but contained other predefined focal-block terms, such as perceived competence, together with a mandatory well-being term. Annual trajectories through 2025 are shown in Figure 11. Among the four critical-thinking records, one treated the construct as a primary focus, one as a secondary outcome, and two used it only in incidental framing; the 1.9% figure should not be interpreted as four equivalent substantive studies.

### 3.10. Synthesis of the Retrieved Corpus

Across performance and science-mapping indicators, motivation and self-determination theory were substantially more consolidated than self-efficacy, creativity, or critical thinking. This pattern is a property of the retrieved intersectional corpus and does not establish that the less frequent constructs are unimportant or absent from the wider Physical Education literature.

## 4. Discussion

### 4.1. General Interpretation of the Findings and Relevance to Sport Pedagogy

This study describes 206 documents published between 2008 and 2026 at the intersection defined by the search equation. Annual production through 2025 increased substantially, but the pattern is descriptive and the bibliometric data alone cannot establish that pedagogical change caused the increase. Spain led corresponding-author output, while Australia showed the highest MCP proportion among the leading countries.

The distribution of sources and collaborations indicates that the retrieved literature crosses Physical Education pedagogy, psychology, public health, and educational research. This interdisciplinarity should be interpreted cautiously because global citation and publication patterns are also shaped by database coverage, journal reach, and disciplinary audience.

The re-screening and subsequent targeted eligibility audit materially changed the evidence base by removing clinical patient education, non-educational sport and coaching, general physical-activity programmes, broad motivation theories, and other records that did not satisfy the operational scope. Consequently, the present results replace all interpretations based on the original 475-record dataset.

### 4.2. Motivation as a Dominant Axis in Physical Education

Motivation was explicitly represented in 84.5% of documents and had one of the highest PageRank values. Self-determination theory, autonomy support, and basic psychological needs were also prominent in the author-keyword structure. Within this retrieved corpus, motivation therefore constitutes the most consolidated focal line.

This result is consistent with the strong influence of self-determination theory in Physical Education research. According to Deci and Ryan (1985) and Ryan and Deci (2000), students are more likely to develop self-determined forms of motivation when their basic psychological needs for autonomy, competence, and relatedness are satisfied. In Physical Education, these needs are particularly relevant because students’ engagement depends not only on the content taught, but also on how tasks are structured, how feedback is provided, how social relationships are promoted, and how competence is experienced (Ntoumanis, 2001; Standage et al., 2005; Vasconcellos et al., 2020).

The pattern is consistent with the pedagogical importance of engagement, enjoyment, effort, perceived competence, and sustained participation (Ntoumanis, 2001; Standage et al., 2005; Vasconcellos et al., 2020; Wallhead & Ntoumanis, 2004). However, co-occurrence centrality indicates structural prominence, not the effectiveness of any instructional strategy or a causal mechanism.

The contrast between motivation and the other focal constructs is best interpreted as relative representation within this search-defined corpus. It does not demonstrate that creativity or critical thinking are globally underdeveloped across all Physical Education research.

### 4.3. Self-Efficacy and Perceived Competence

Self-efficacy appeared in 34 documents (16.5%) and in 11 titles or author-keyword sets (5.3%). This representation is compatible with theory linking capability beliefs to participation, persistence, and recovery from failure (Bandura, 1977; Moritz et al., 2000), but the bibliometric analysis does not estimate the strength of those relationships.

Perceived competence also intersects with self-determination theory as a basic psychological need (Ryan & Deci, 2000). Pedagogical approaches such as differentiated tasks, formative feedback, or autonomy support may be relevant according to prior educational literature, but their effectiveness is not tested by the present maps.

### 4.4. Creativity and Critical Thinking: Limited Representation in the Retrieved Corpus

Creativity was explicit in 16 documents (7.8%) and salient in seven (3.4%); critical thinking appeared explicitly in four (1.9%) and saliently in only one (0.5%). Neither formed an independent thematic cluster or reached the three-document author-keyword threshold. These figures establish relative underrepresentation in this corpus, not an educational deficit by themselves.

Independent pedagogical theory nevertheless provides reasons to examine both constructs: creativity may involve movement exploration, tactical alternatives, bodily expression, and open-ended problem solving (Beghetto & Kaufman, 2007; Runco & Jaeger, 2012), while critical thinking may involve reasoned tactical analysis, peer assessment, health literacy, and reflection (Halpern, 1998). The combination of theoretical relevance and low corpus representation identifies a research opportunity rather than proving a field-wide gap.

Future empirical studies could test whether specific pedagogical models foster these outcomes and should define them explicitly, use validated measures, and distinguish primary outcomes from incidental mentions. Such proposals derive from the bibliometric pattern interpreted alongside pedagogical literature, not from evidence of intervention effectiveness in the present study.

### 4.5. Positive Psychological Perspectives as an Integrative Framework

Positive Psychology did not emerge as a dominant independent cluster. Instead, well-being, mental health, subjective well-being, and related terms were distributed across the network. Positive psychological perspectives are therefore more defensibly treated as an interpretive lens that may connect otherwise separate lines than as the empirically established organising framework of the field.

Positive Psychology focuses on strengths, positive emotions, engagement, relationships, meaning, accomplishment, and well-being (Seligman & Csikszentmihalyi, 2000; Seligman et al., 2009). In education, it has been associated with resilience, engagement, social connection, and personal growth (Kern et al., 2015; Norrish et al., 2013; Waters, 2011). Physical Education offers a suitable context for applying this framework because it combines bodily experience, social interaction, emotional regulation, challenge, achievement, cooperation, and self-perception.

This distinction is important: the search required well-being or positive-psychological terminology, whereas the resulting conceptual structure was more strongly organised around motivation and self-determination theory. The maps thus show incomplete integration rather than confirmation of a single umbrella theory.

### 4.6. Implications for Sport Pedagogy and Practice

The findings support cautious agenda-setting rather than direct prescriptions for practice. The prominence of motivation and competence-related terms suggests that autonomy, competence, relatedness, and student experience remain important topics for pedagogical research.

Based on prior pedagogical literature, not on effectiveness estimates from this bibliometric study, future intervention research may examine differentiated tasks, formative feedback, cooperative structures, open-ended motor problems, and reflective questioning. These strategies require direct empirical testing with clearly specified outcomes.

Similarly, positive-education practices should not be presented as consequences of the maps. Their relevance lies in providing testable hypotheses about how Physical Education might support well-being, confidence, relationships, and agency across school, university-course, and teacher-education contexts.

### 4.7. Limitations of the Study

This study presents several limitations that should be acknowledged. First, the analysis was based exclusively on documents indexed in Scopus and Web of Science Core Collection. Although these databases are widely recognised and commonly used in bibliometric research, relevant studies indexed in other databases such as ERIC, PubMed, SportDiscus, PsycINFO, or Google Scholar may have been excluded.

The search was limited to English- or Spanish-language records in Scopus and Web of Science and required a term from each of three Boolean blocks. Consequently, it maps an intersectional corpus rather than all research on the four constructs in Physical Education. Relevant studies using other terminology or indexed elsewhere may have been missed. Conversely, broad terms such as PE initially produced retrieval noise; the complete re-screening reduced but cannot eliminate all classification uncertainty. Full texts were consulted when metadata were insufficient. Metadata variation in authors, affiliations, keywords, and cited references may also affect results.

The analysis does not assess methodological quality, theoretical depth, or intervention effectiveness. Citation counts represent global bibliometric visibility and remain sensitive to publication age, journal audience, and disciplinary differences; normalisation reduces but does not remove these effects. Keyword classifications also depend on author and database terminology, and construct categories overlap.

### 4.8. Future Research

Future research should test theoretically justified relationships among motivation, self-efficacy, creativity, critical thinking, and well-being using designs suited to causal or developmental questions. Creativity and critical thinking should be operationalised as primary or secondary outcomes rather than inferred from incidental wording.

Comparative work across school Physical Education, university Physical Education courses, and initial Physical Education teacher education could clarify contextual differences. Broader systematic searches that do not require the well-being block would also help determine whether the distribution observed here is specific to the present intersectional strategy.

## 5. Conclusions

This revised bibliometric study mapped 206 eligible documents published between 2008 and 2026. Motivation was substantially more represented than self-efficacy, creativity, or critical thinking. The DE-only network and thematic map were organised primarily around Physical Education, motivation, well-being, physical activity, and self-determination theory; creativity and critical thinking did not form prominent clusters.

These results are a descriptive map of the corpus generated by the three-block search strategy. Positive Psychology is best treated as a possible integrative lens rather than the field’s demonstrated dominant framework, and lower keyword frequency should not be equated with lower educational importance. The revised dataset, transparent screening record, and construct-specific analyses provide a more defensible basis for future empirical work.

## Figures and Tables

**Table 1:** Conceptual blocks and search terms.

Conceptual Block	Search Terms
Physical Education	“physical education” OR “school physical education” OR “PE” OR “physical activity education”
Positive Psychology and well-being	“positive psychology” OR wellbeing OR “well-being” OR “positive education” OR “social-emotional learning” OR “emotional education”
Psychological and cognitive constructs	creativity OR “creative thinking” OR “critical thinking” OR self-efficacy OR “perceived competence” OR motivation OR “intrinsic motivation” OR “self-determined motivation”

**Table 2:** Database-specific adaptation of the search strategy.

Database	Search Field	Database-Specific Search Equation
Scopus	TITLE-ABS-KEY	TITLE-ABS-KEY ((“physical education” OR “school physical education” OR “PE” OR “physical activity education”) AND (“positive psychology” OR wellbeing OR “well-being” OR “positive education” OR “social-emotional learning” OR “emotional education”) AND (creativity OR “creative thinking” OR “critical thinking” OR self-efficacy OR “perceived competence” OR motivation OR “intrinsic motivation” OR “self-determined motivation”))
Web of Science Core Collection	TS	TS = ((“physical education” OR “school physical education” OR “PE” OR “physical activity education”) AND (“positive psychology” OR wellbeing OR “well-being” OR “positive education” OR “social-emotional learning” OR “emotional education”) AND (creativity OR “creative thinking” OR “critical thinking” OR self-efficacy OR “perceived competence” OR motivation OR “intrinsic motivation” OR “self-determined motivation”))

**Table 3:** Most productive countries according to corresponding-author affiliation. Article percentages use the 202 records with an identifiable corresponding-author country.

Rank	Country	Articles	Article %	SCP	MCP	MCP %
1	Spain	48	23.8	43	5	10.4
2	USA	23	11.4	20	3	13.0
3	China	14	6.9	11	3	21.4
4	Australia	10	5.0	3	7	70.0
5	United Kingdom	9	4.5	4	5	55.6
6	Turkey	8	4.0	4	4	50.0
7	Italy	7	3.5	5	2	28.6
8	Norway	7	3.5	5	2	28.6
9	Germany	5	2.5	5	0	0.0
10	Iran	5	2.5	3	2	40.0

**Table 4:** Most frequent institutional affiliations.

Rank	Affiliation	Occurrences
1	University of Almería	20
2	University of Newcastle	19
3	University of Castilla-La Mancha	14
4	University of Wyoming	12
5	Ghent University	11
6	University of Murcia	10
7	University of Queensland	10
8	University of Valencia	10
9	University of Granada	9
10	University of Tabriz	9

**Table 5:** Most relevant sources according to publication output.

Rank	Source	Documents
1	Frontiers In Psychology	15
2	Physical Education And Sport Pedagogy	13
3	Journal Of Teaching In Physical Education	11
4	Bmc Public Health	7
5	European Physical Education Review	7
6	International Journal Of Environmental Research And Public Health	7
7	Children-Basel	5
8	Journal Of Sport & Exercise Psychology	5
9	Retos-Nuevas Tendencias En Educacion Fisica Deporte Y Recreacion	5
10	Behavioural Sciences	4

**Table 6:** Most productive authors.

Rank	Author	Documents	Fractionalised Articles
1	Simonton K	10	2.47
2	Gaudreault K	6	1.33
3	Richards K	6	1.60
4	Behzadnia B	5	2.33
5	Lubans D	5	0.81
6	Mercier K	5	0.98
7	Trigueros R	5	1.40
8	Aguilar-Parra J	4	0.90
9	Granero-Gallegos A	4	1.03
10	Lonsdale C	4	0.75

**Table 7:** Most globally cited documents within the retrieved corpus.

Rank	Paper	DOI	Total Citations	TC per Year	Normalised TC
1	Standage et al. (2012), J Sport Exerc Psychol	10.1123/jsep.34.1.37	264	17.60	1.77
2	Mouratidis et al. (2008), J Sport Exerc Psychol	10.1123/jsep.30.2.240	236	12.42	1.00
3	Haerens et al. (2018), Phys Educ Sport Pedag	10.1080/17408989.2017.1346070	161	17.89	4.14
4	Cheon et al. (2014), J Sport Exerc Psychol	10.1123/jsep.2013-0231	154	11.85	3.98
5	Behzadnia et al. (2018), Psychol Sport Exerc	10.1016/j.psychsport.2018.07.003	137	15.22	3.52
6	Warburton et al. (2020), Motiv Emot	10.1007/s11031-019-09798-2	108	15.43	4.63
7	González-Cutre and Sicilia (2019), Eur Phys Educ Rev	10.1177/1356336X18783980	85	10.62	2.64
8	Sierra-Díaz et al. (2019), Front Psychol	10.3389/fpsyg.2019.02115	78	9.75	2.42
9	Luna et al. (2019), Int J Environ Res Public Health	10.3390/ijerph16101821	64	8.00	1.99
10	Liu et al. (2017), School Ment Health	10.1007/s12310-017-9223-6	63	6.30	2.25

**Table 8:** Representation of the four focal constructs in the final corpus.

Construct	Explicit Documents	Explicit %	Title/Author-Keyword Documents	Title/Author-Keyword %
Motivation	174	84.5	76	36.9
Self-efficacy	34	16.5	11	5.3
Creativity	16	7.8	7	3.4
Critical thinking	4	1.9	1	0.5

## Data Availability

The complete list of the 206 included documents is provided as File S1. The cleaned 206-record bibliographic dataset is supplied as CSV (File S2) and BibTeX (File S3), and the document-level construct classification is supplied as CSV (File S4).

## Supplementary Materials

The following supporting information can be downloaded at: https://www.mdpi.com/article/10.3390/bs16091673/s1.
